# A coherent synchrotron X-ray microradiology investigation of bubble and droplet coalescence

**DOI:** 10.1107/S0909049508025363

**Published:** 2008-10-03

**Authors:** B. M. Weon, J. H. Je, Y. Hwu, G. Margaritondo

**Affiliations:** a X-ray Imaging Center, Department of Materials Science and Engineering, Pohang University of Science and Technology, Pohang 790-784, Korea; b Institute of Physics, Academia Sinica, Nankang, Taipei 11529, Taiwan; c Ecole Polytechnique Fádárale de Lausanne (EPFL), CH-1015 Lausanne, Switzerland

**Keywords:** coherent synchrotron X-ray, microradiology, coalescence, bubbles, droplets

## Abstract

A quantitative application of microradiology with coherent X-rays to the real-time study of microbubble and microdroplet coalescence phenomena, with specific emphasis on the size relations in three-body events, is presented. The results illustrate the remarkable effectiveness of coherent X-ray imaging in delineating interfaces in multiphase systems, in accurately measuring their geometric properties and in monitoring their dynamics.

## Abstract 2

Microradiology with coherent X-rays is shown to be very effective in revealing interfaces in multiphase systems and in particular gas bubbles. Its use has been tested in the study of bubble colescence validating the results with a simple theoretical analysis based on mass conservation.

## 1. Introduction

Bubbles and droplets are very interesting systems because of their fundamental properties and practical applications; this is particularly true for their coalescence (Illingworth, 1988 ▶; Trizac & Hansen, 1995 ▶; Eggers *et al.*, 1999 ▶; Bowker, 2002 ▶; Fialkowski *et al.*, 2005 ▶; Aarts *et al.*, 2005 ▶; Maris & Balibar, 2005 ▶; Yao *et al.*, 2005 ▶; Winterhalter & Sonnen, 2006 ▶; Ristenpart *et al.*, 2006 ▶; Daniel *et al.*, 2001 ▶; Hawa & Zachariah, 2006 ▶; Atencia & Beebe, 2005 ▶; Whitesides, 2006 ▶; Janasek *et al.*, 2006 ▶). It is not easy, however, to accurately monitor dynamic properties on a microscopic scale. Here we show that microradiology with the spatially coherent X-rays emitted by a synchrotron source (Snigirev *et al.*, 1995 ▶; Nugent *et al.*, 1996 ▶; Wilkins *et al.*, 1996 ▶; Cloetens *et al.*, 1996 ▶; Tsai *et al.*, 2002 ▶; Baik *et al.*, 2004 ▶; Margaritondo *et al.*, 2004 ▶; Weon *et al.*, 2006 ▶) can be very effective in this context. This technique dynamically detects the boundaries of very small bubbles and droplets and makes it possible to measure geometric properties with high accuracy. The same is valid in general for the gas–liquid interfaces that control many of the interesting phenomena in multiphase fluid dynamics.

## 2. Experimental and discussion

**Figure 1:**
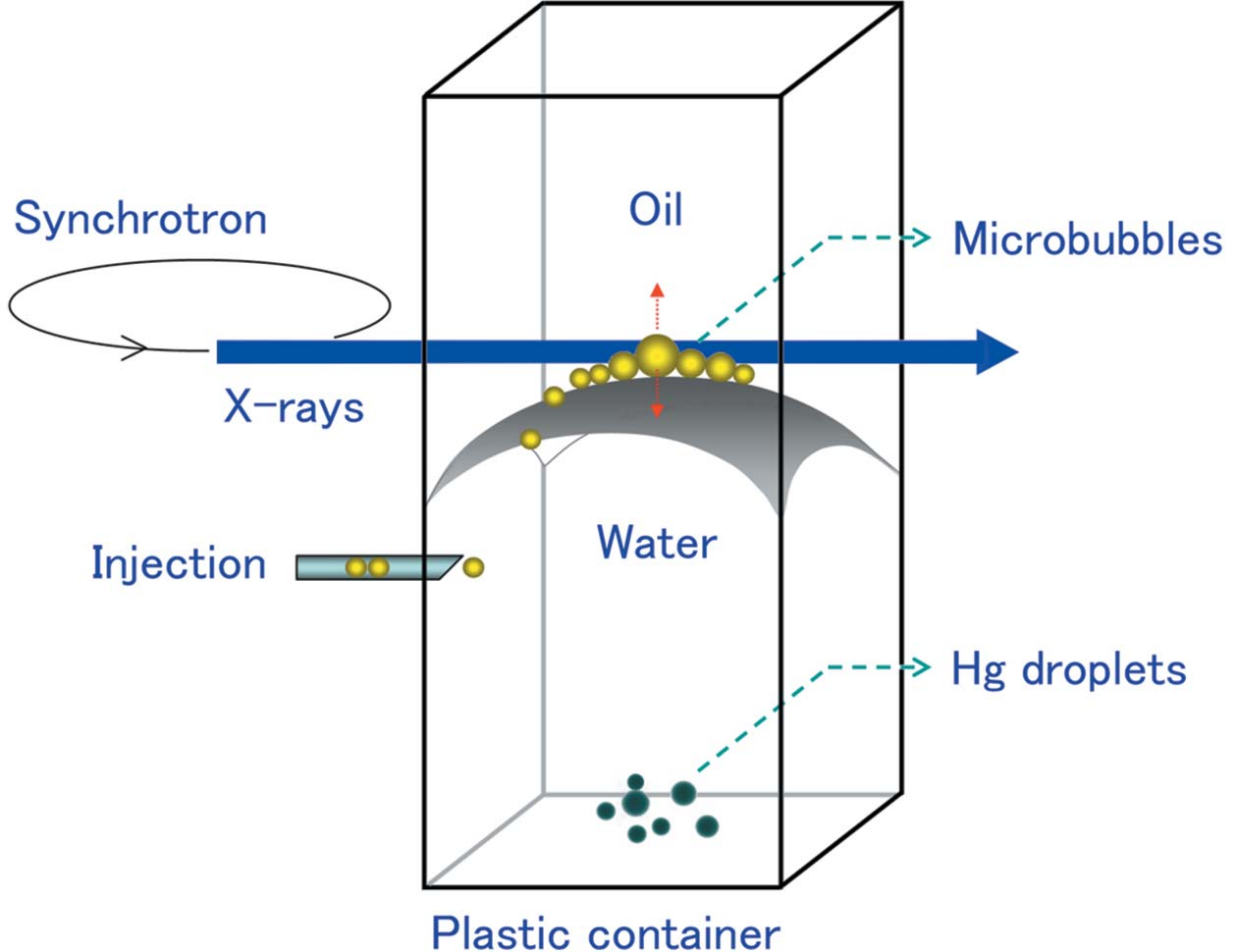
Experimental set-up for the observation of coalescence phenomena for gas of microbubbles and mercury microdroplets. The capillary properties of the water–oil interface in a plastic container countered the gravity effects on the microbubbles.

We specifically analyzed coalescence events involving gas bubbles or mercury droplets. For bubbles, we exploited the capillary properties of the water–oil interface in a plastic container (10 × 10 × 100 mm) (Fig. 1 ▶) to largely suppress the influence of gravity and of the liquid. After injection into water, the microbubbles are confined to move at the water–oil interface; they shift towards its center-top because of the net force resulting from the (vertical) gravitation buoyancy combined with the adhesion force (perpendicular to the interface). At the center-top of the interface, they coalesce together and the events are recorded with sequential real-time microradiographs.

**Figure 2:**
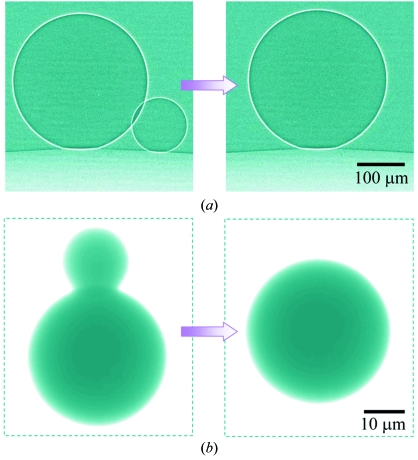
Sequential images of coalescence events taken in real time using synchrotron X-ray microradiography: (*a*) two coalescing air microbubbles at the water–oil interface and (*b*) mercury microdroplets in water.

Overall, the microbubbles at our water–oil interface are similar to those in reduced gravity (Weaire, 2002 ▶; Hilgenfeldt, 2002 ▶; Divinis *et al.*, 2004 ▶): they have almost spherical shapes, and adjacent microbubbles have point contacts rather than flat contact planes and merge into bigger microbubbles without drainage (Fig. 2*a* ▶). These similarities, however, are present only for sufficiently small microbubbles: specifically, near-sphericity occurs when gravitational effects are negligible with respect to the surface tension effects. This is true if *B*_0_ → 0, where *B*_0_ = ρ_D_ *gr* ^2^/γ is the Bond number, ρ_D_ is the water–oil density difference and *g* is the gravity acceleration (Aarts *et al.*, 2005 ▶; Divinis *et al.*, 2004 ▶). We empirically found deviations from sphericity (relative difference between the vertical and horizontal diameters) of 3–4% for bubble diameters of 400 µm and 0–1% for diameters of 100 µm. Thus, quantitative studies must be preferentially performed on microbubbles of diameter < 400 µm.

**Figure 3:**
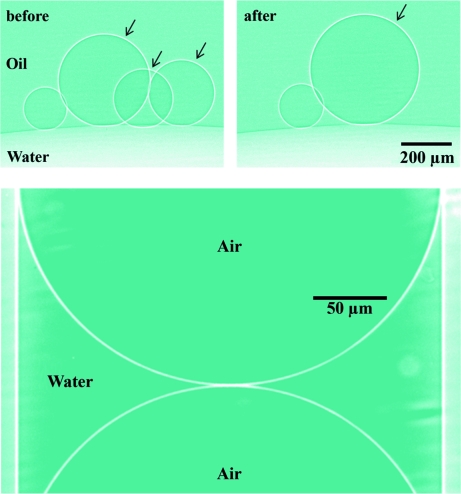
Top: a three-bubble coalescence event. Bottom: air–water interfaces for bubbles in a capillary tube.

Phase-contrast microradiography was implemented with un­monochromatized coherent synchrotron X-rays in the photon energy range 10–60 keV (from the PLS 7B2 beamline in Pohang, Korea). Figs. 2 ▶ and 3 ▶ illustrate interfaces delineated with remarkable sharpness. Specifically, Fig. 2 ▶ shows two-particle coalescence events for gas bubbles and mercury droplets, and Fig. 3 ▶ shows a three-bubble coalescence event as well as the sharp air–water interfaces of bubbles in a capillary tube.

Our size measurements were validated by their consistency with mass conservation. Calling ρ the density and *r* the particle radius, a particle mass *m* equals 4π*r* ^3^ρ/3. For two-particle coalescence, *m*_m_ = *m*_s_ + *m*_l_, where *m*_s_ and *m*_l_ refer to the smaller and larger coalescing particles and *m*_m_ to the final product. Considering the Young–Laplace equation, *p* − *p*_0_ = 2γ/*r* [where *p* − *p*_0_ is the pressure difference between the particle and the surrounding medium, γ is the surface tension, independent of the radius (Onischuk *et al.*, 2006 ▶)], and assuming a linear relation ρ = ρ_0_ + *A*(*p* − *p*_0_), this equation becomes
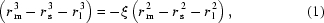
where ξ = 2γ*A*/ρ_0_.

The quadratic part of (1) can be neglected for gas bubbles. In fact, for microbubbles in most liquids we can take a typical value γ ≃ 50 mN m^−1^ and use the ideal-gas limit ξ = 2γ/*p*_0_; with atmospheric-level pressure the ξ values are in the micrometre range, much smaller than the radii, and (1) becomes 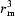 = 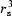 + 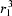.

**Figure 4:**
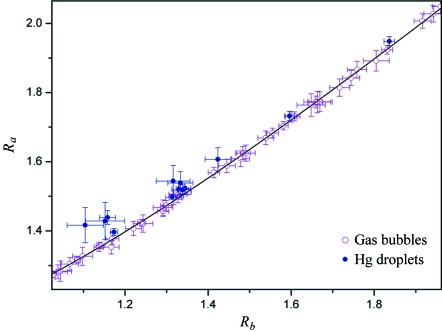
Measured sizes for many different coalescence events plotted in terms of variables *R*_*a*_ = *r*_m_/*r*_s_ and *R*_*b*_ = *r*_l_/*r*_s_. The solid line shows the cubic mass conservation relation (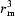 = 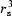 + 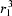). The error bars correspond to a standard deviation in the radius measurements.

Coalescence events were recorded for microbubbles with radii in the range 10–300 µm for different gases (Ar, He and air), different liquid temperatures (between 290 K and 330 K) and different oil–water interface curvatures. Radius values were extracted from the images using *Image-ProPlus* software (MediaCybernetics) and the accuracy was determined by the spatial resolution and, for large bubbles, by deviations from sphericity. The overall trend is shown in Fig. 4 ▶ (open circles) in terms of the variables *R*_*a*_ = *r*_m_/*r*_s_ and *R*_*b*_ = *r*_l_/*r*_s_. The best fit (solid line), independent of the gas, liquid temperature and interface curvature, corresponds to the above cubic form, 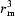 = 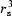 + 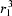 (Bolina & Parreira, 2000 ▶).

As for mercury droplets, after injection in water they go down to the bottom of the plastic container where coalescence takes place. As seen in Fig. 4 ▶ (full dots), the experimental points do not seem entirely consistent with a purely cubic relation. This should be explained by the complete form of the mass conservation, equation (1).

## 3. Conclusion

In summary, we have used coherence-based synchrotron microradiology to image coalescence phenomena involving gas bubbles in a microgravity-like environment and mercury droplets. This technique was very effective in delineating interfaces in these multiphase systems and enabled us to measure radii with micrometre-level accuracy; simple mass conservation arguments validated the results. In general terms, our images clearly illustrate the potential of coherence-based contrast in accurate studies of the dynamics of multiphase systems.
